# Identification for Differential Localization of Putative Corneal Epithelial Stem Cells in Mouse and Human

**DOI:** 10.1038/s41598-017-04569-w

**Published:** 2017-07-12

**Authors:** Jin Li, Yangyan Xiao, Terry G. Coursey, Xin Chen, Ruzhi Deng, Fan Lu, Stephen C. Pflugfelder, De-Quan Li

**Affiliations:** 1 Zhejiang Eye Hospital, School of Optometry and Ophthalmology, Wenzhou Medical University, Wenzhou, China; 2 Department of Ophthalmology, the Second Xiangya Hospital, Central South University, Changsha, China; 3 Ocular Surface Center, Cullen Eye Institute, Department of Ophthalmology, Baylor College of Medicine, Houston, Texas USA

## Abstract

Human Corneal epithelial stem cells (CESCs) have been identified to reside in limbus for more than 2 decades. However, the precise location of CESCs in other mammalian remains elusive. This study was to identify differential localization of putative CESCs in mice. Through a series of murine corneal cross-sections from different directions, we identified that anatomically and morphologically the murine limbus is composed of the thinnest epithelium and the thinnest stroma without any palisades of Vogt-like niche structure. The cells expressing five of stem/progenitor cell markers are localized in basal layer of entire murine corneal epithelium. BrdU label-retaining cells, a key characteristic of epithelial stem cells, are detected in both limbal and central cornea of mouse eye. Functionally, corneal epithelium can be regenerated in cultures from central and limbal explants of murine cornea. Such a distribution of mouse CESCs is different from human cornea, where limbal stem cell concept has been well established and accepted. We are aware that some new evidence supports limbal stem cell concept in mouse recently. However, it is important to know that central cornea may provide an alternative source of stem cells when one utilizes mice as animal model for corneal research.

## Introduction

Integrity of corneal epithelium is important for corneal transparency and vision. The corneal epithelium, which composed of superficial layers of flattened cells called squamas, layers of suprabasal or wing cells, and a single layer of columnar basal cells, is regenerated throughout life by corneal epithelial stem cells (CESCs), which are known as the reservoir responsible for maintaining the homeostasis of corneal epithelium. Human CESCs have been identified to be located in the basal epithelial layer of the limbus, a 1.5 mm to 2 mm wide area that straddles the cornea and bulbar conjunctiva. Substantial evidence from a large amount of investigations in last two decades leaves little doubt that human CESCs reside in the limbus and exhibit the full complement of well-defined keratinocyte stem cell properties, including the lack of the K3/K12 keratin pair in limbal basal cells, the existence of label-retaining cells at this location, their higher proliferative potential compared with central corneal cells, and their ability to grow in colony-forming assays^1–5^. Thus, CESCs are also referred to as limbal stem cells (LSC) based on their location.

The LSC hypothesis is based on XYZ theory of corneal epithelial homeostasis. X represents proliferation and stratification of limbal basal cells; Y, centripetal migration of basal cells; and Z, desquamation of superficial cells^6^. Clinically, limbal stem cell deficiency (LSCD), a frequently encountered problem, has been recognized as a sight threatening disease that may causes blindness, and the great progress has been achieved using limbal stem cell transplantation and other therapy based on LSC concept^7–13^. Our previous studies also provide strong evidence supporting LSC concept in human^14–16^.

However, the anatomical location of CESCs in different mammalian species is still controversial and remains elusive. Majo and colleagues proposed an alternative hypothesis in 2008 that murine CESCs are distributed throughout the basal layer of entire corneal epithelium because central corneal epithelium could contribute to long-term self-renewal and be capable of sustaining serial transplantation^17^. They demonstrated that the stem cells in the cornea were responsible for regeneration of central corneal epithelium while limbal stem cells mainly for limbal epithelial repair. Destruction of entire limbal stem cells by severe burn did not disturb the transparency of murine cornea, suggesting that steady-state renewal of cornea did not depend only on limbal stem cells.

Later Notara group has demonstrated the common structures with similar phenotype and function in the porcine and human limbus in terms of the location, topography, stem cell markers and proliferative capacity of palisades of Vogt^18^. Very recently, Patruno *et al*. investigated the main distinctive structural features of the corneal limbus in a variety of animal species including dog, cat, pig, cow, sheep and horse, using optic microscope observations of histological sections. Their data revealed that the limbal epithelium of the horse and pig showed similarities with human corneal epithelium in terms of structure and ΔNp63α expression^19^.

However, little data have shown the anatomical structure of limbus and the phenotype of the central cornea in mice. The present study provides new evidence indicating that murine corneal stem cells are localized in the entire cornea, but not limited to limbal area, based on structural morphology, expression and location of stem/progenitor cell markers, label-retaining cells, and proliferative capacity of central corneal epithelium in murine cornea, a differential phenotype from human corneal epithelium.

## Results

### Anatomical structures of murine corneal limbus differ from human limbus

**Figure 1:**
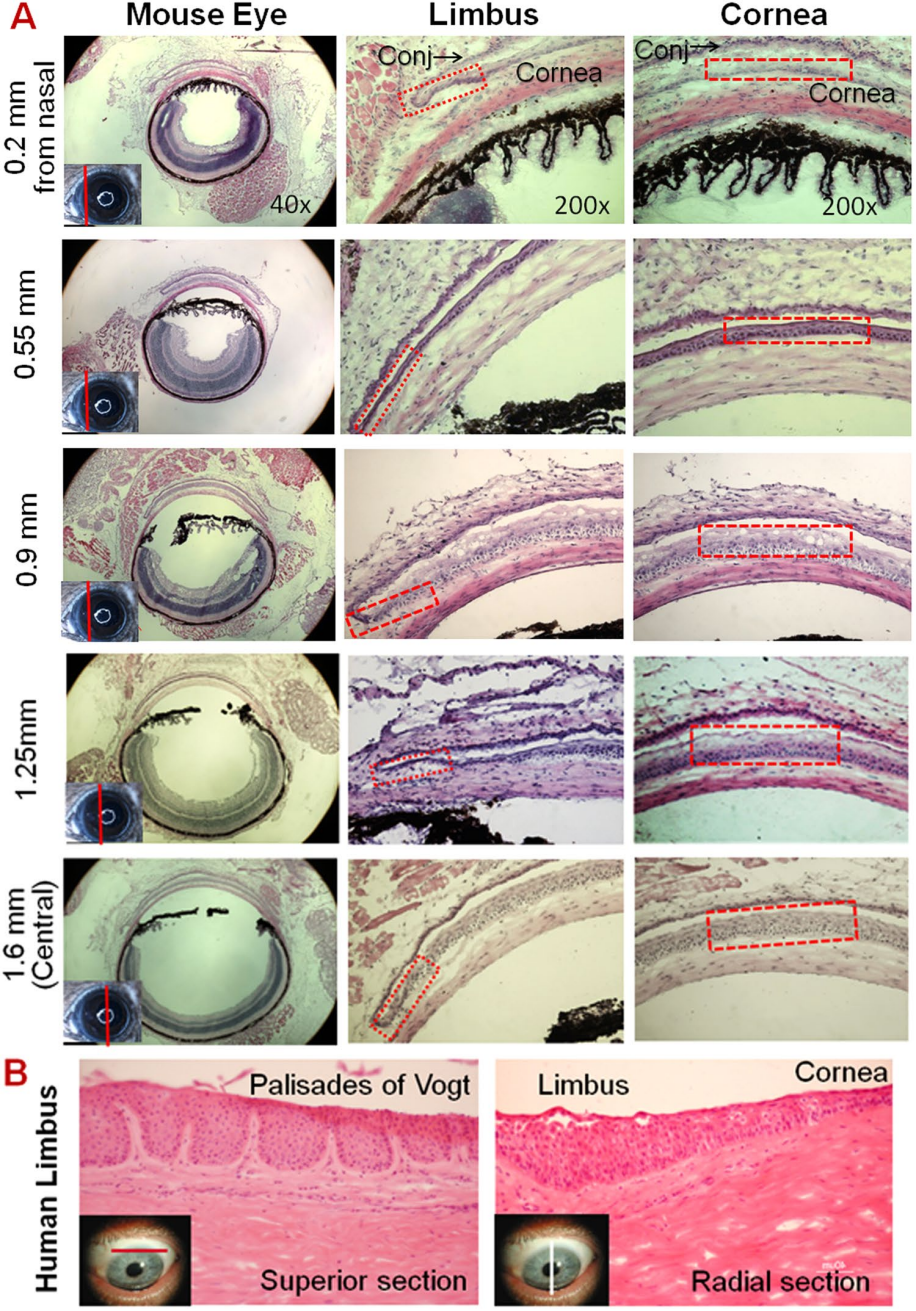
Representative Haematoxylin-Eosin (H & E) staining images of mouse and human corneas. (**A**) Five sets of images were chosen from a series of mouse corneal cross sections from nasal to central cutting. The left column shows images of whole eye sections at different positions as red line on small eye inserts. The middle column shows limbal structures with red rectangles indicating the limbal epithelium containing 1~3 layers of cells. The right column shows corneal structures with red rectangles indicating the 5–7 layers of central corneal epithelium. (**B**) Human limbal sections with vertical or horizontal cutting show the palisades of Vogt structures.

To thoroughly examine the anatomical structures of mouse cornea, we performed hematoxylin and eosin staining (H & E) on serial cross sections of C57BL/6 mouse eyes (10 mice) through radial and tangential cut, as well as different directions from superior to inferior and from nasal to temporal. In Fig. 1A, the representative images of 5 positions of cross sections, which were separated by approximately 0.35 mm each other from 0.2 mm to nasal to central cornea based on a schematic measurement of mouse eye^20^, represented, from left to right, the cross sections of the entire eye, peripheral/limbal cornea and the central cornea. We observed that mouse central corneal epithelium was the thickest area containing 5–7 (5.8 ± 1.2, n = 10) layers of epithelial cells, similar to human central cornea that contains 5–8 (6.3 ± 1.6, n = 10, *P* > 0.05) epithelial layers. However, the murine corneal epithelium became thinner from central to peripheral areas, and the limbal margin was the thinnest area containing only 1–3 (1.8 ± 0.8, n = 10) epithelial layers, which was totally different from human limbus that contains 7–10 (8.3 ± 1.8, n = 10, *P* < 0.001) layers of epithelial cells. Human limbal epithelium became much thicker when transitioned from peripheral cornea. Human limbus is the thickest area of epithelium with densely packed small stem/progenitor cells in the basal layer of papilla-like epithelial column, together with multiple stromal invaginations forming a special niche structure called the palisades of Vogt (Fig. 1B)^14, 21^. There were also blood vessels, nerves and connective tissue between the epithelial columns. The microenvironment of the niche structure is essential to maintaining quiescence of stem cells, while also providing protection from mechanical and chemical injures. As seen in Fig. 1A, murine limbal epithelium was a thinner margin without such anatomical structures serving as stem cell niche as found in the human cornea.

### Cells expressing stem/progenitor cell markers are localized in basal layer of entire murine corneal epithelium

**Figure 2:**
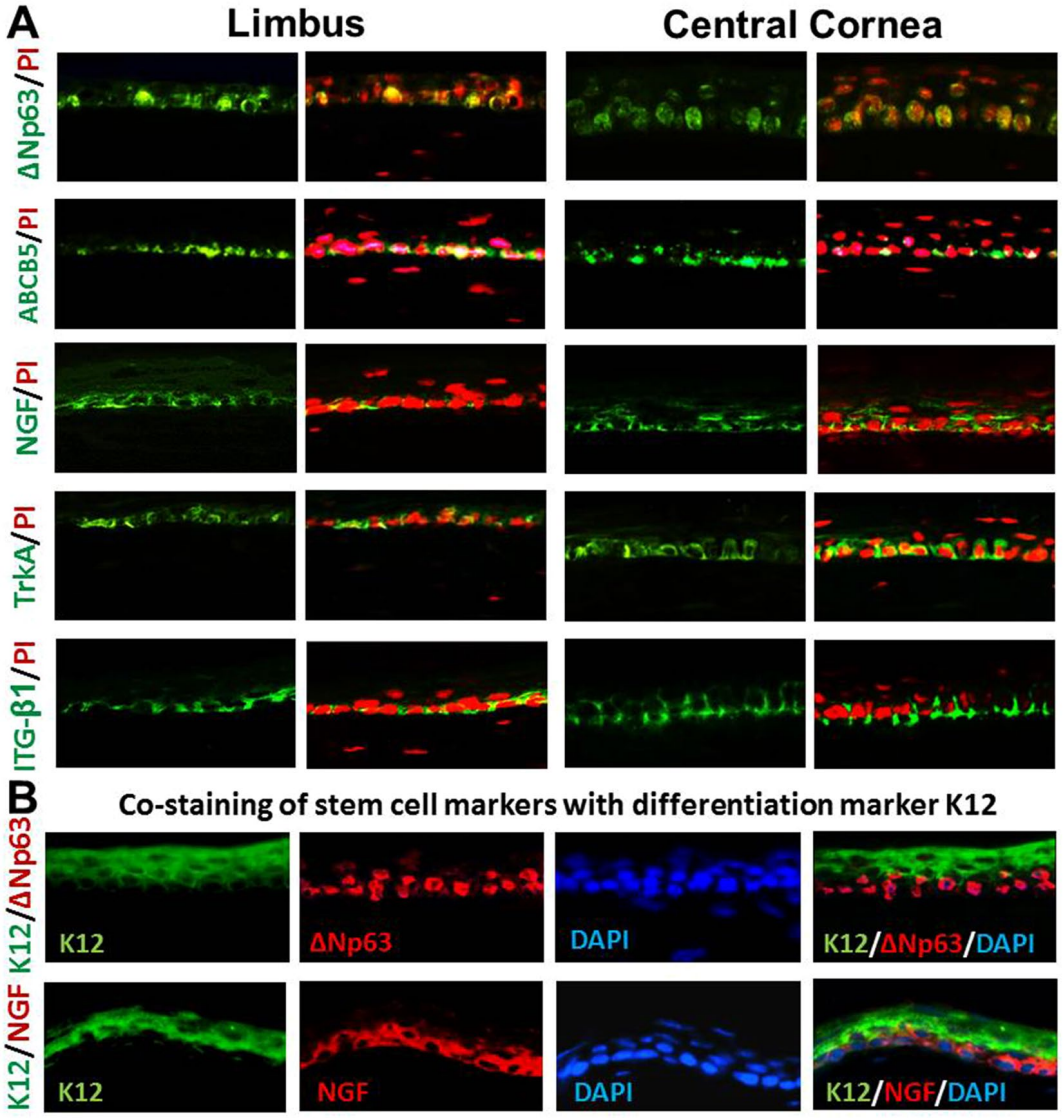
Representative images of Immunofluorescent staining of stem cell markers. (**A**) Five proposed stem/progenitor cell markers, ΔNp63, ABCB5, NGF, TrkA and integrin β1 (ITG-β1), were mainly expressed by some basal epithelial cells in both limbus and central cornea. (**B**) Double staining with corneal specific marker K12 and stem cell markers ΔNp63 and NGF. Image magnification: 400×.

A group of molecular markers has been used for identification of human corneal stem/progenitor cells due to no definitive markers exist for adult stem cells to date^14, 22, 23^. Many molecular markers are expressed exclusively or significantly stronger in some human limbal basal cells than central corneal epithelium. This study used the same strategy for human CESCs to identify the potential stem/progenitor cells localized in murine cornea. These markers were ΔNp63, a isoform of nuclear transcription factor p63^12^, ATP-binding cassette protein-B5 (ABCB5)^24^, integrin-β1^25^, nerve growth factor (NGF) and its receptor TrkA^26^. The immunofluorescent staining showed that these five markers were expressed by some cells in the basal layer of entire corneal epithelium including central and peripheral/limbal areas in murine cornea, although the type, intensity, frequency and distribution pattern of these markers in positive cells were not equal (Fig. 2A). These results suggest that potential stem/progenitor cells of murine corneal epithelium are not restricted to reside in limbus, but distributed throughout the entire corneal epithelial basal layer. Double staining with corneal specific marker K12 and stem cell markers further showed that murine corneal basal epithelial cells that were positive to ΔNp63 and NGF did not express the differentiation marker K12 (Fig. 2B).

### BrdU label-retaining cells (LRCs) are detected in both limbal and central cornea of mouse eye

**Figure 3:**
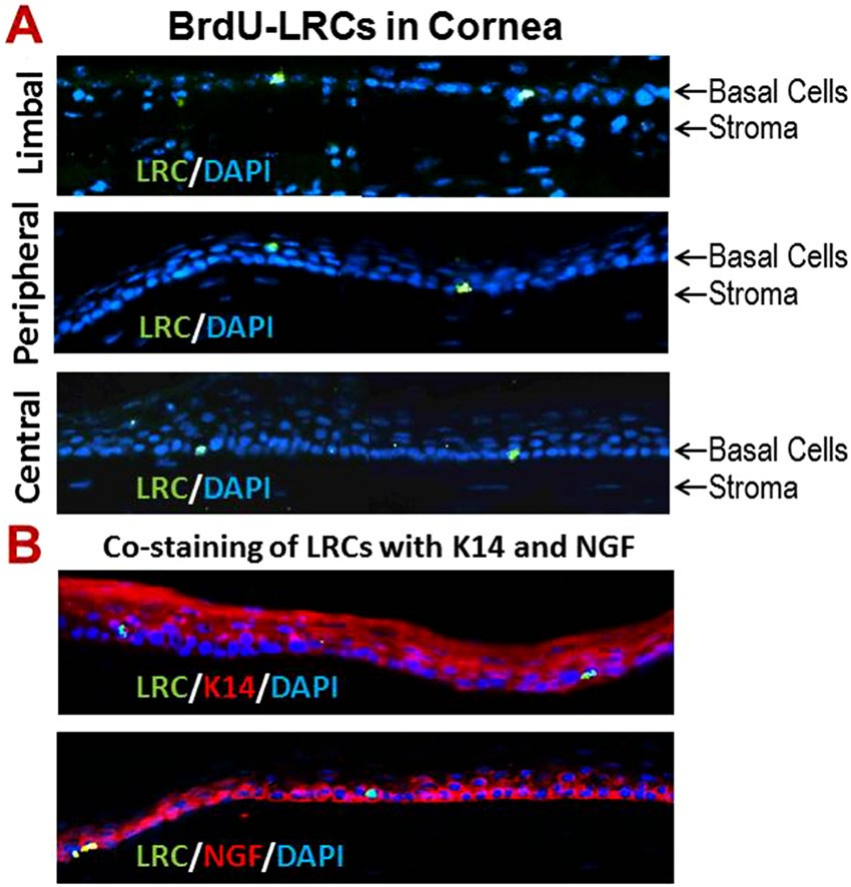
Label- retaining cells (LRCs) detected by BrdU immunofluorescent staining. (**A**) A number of slow cycling LRCs were detected in limbal (top panel), peripheral (middle panel) and central cornea (bottom panel) in C57BL/6 mice with 4 weeks chasing after BrdU injections. See method for detail. (**B**) Double staining of LRCs with epithelial cell marker K14 and stem cell marker NGF. Image magnification: 400×.

The LRCs have been widely accepted as a key characteristic of epithelial stem cells based on that stem cells from adult tissue are quiescently in a growth-arrested or slow cycling state^3^. We sought to detect CESCs by determining if slow cycling BrdU-LRCs are present in murine corneal epithelium. To accomplish this, a standard “pulse-chase” nuclear label retention experiment was performed. Four weeks old C57BL/6 mice were injected with BrdU (1 mg/mouse/day) daily for two weeks and allowed to clear BrdU for four weeks. After the four week chase period, eyes were harvested and the presence of BrdU positive cells was examined by staining cryosections with anti-BrdU antibody. BrdU-positive cells that are slow cycling LRCs were observed in the basal layer of the limbal, peripheral and central cornea of mouse eye (Fig. 3A). These BrdU LRCs were co-stained with epithelial cell marker K14, indicating they were belong to epithelial lineage, and LRCs may also co-stained with stem cell marker such as NGF (Fig. 3B). These findings suggest that CESCs not only exist at the limbus but also reside in the area outside of limbal epithelium in mouse eye. The frequency of LRCs was distributed as 2.6 ± 1.4 in limbus vs. 3.2 ± 1.8 (n = 10, *P* > 0.05) in cornea per mouse eye section, which showed no statistical significance of difference.

### Corneal epithelium can be regenerated in cultures from both central and limbal explants of murine cornea

**Figure 4:**
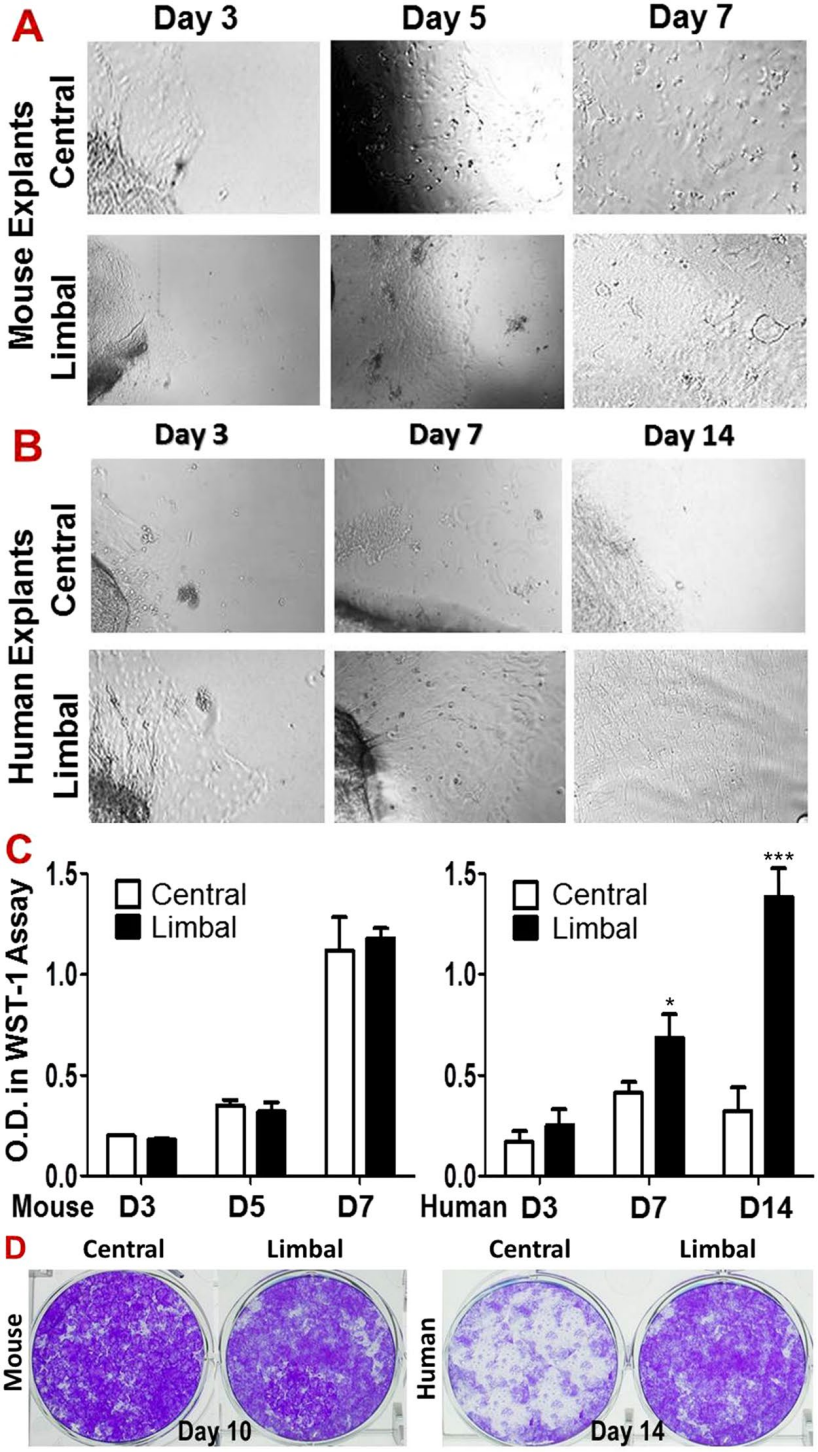
Comparison of proliferative capacity in primary corneal epithelial cultures between limbal and central cornea in mouse and human. (**A**) Phase-contrast microscopy showed the mouse epithelial cell growth of explants from limbal and central corneas on days 3, 5 and 7; (**B**) Human epithelial cell growth of explants from limbal and central corneas on days 3, 7 and 14. (**C**) WST-1 assay showed no difference of epithelial cell growth between limbal and central corneal explants from mice during 7 days (n = 5, P > 0.05); but there is growth difference between limbal and central corneal explants of human during 14 days. Data were summarized from 5 separated experiments. *P < 0.05 and ***P < *0.001*, as two groups compared. (**D**) The representative images from clonogenic assays of the single cells from corneal or limbal epithelia in mice and human.

Furthermore, we sought to determine the proliferative capacity of different parts of murine cornea to functionally identify the localization of CESCs through their growth potential in primary corneal epithelial cultures. To accomplish this, the explants at size of 1 × 1 mm from the central or peripheral/limbal cornea were cultured for seven days and observed by phase contrast microscope. Interestingly, all explants from both the central and peripheral/limbal areas of mouse cornea grew well and became confluent after seven days. No significant difference in growth was observed among the explants from different areas of mouse cornea (Fig. 4A). By contrast, cultures with explants taken from the central human cornea could not be established, and only the explants from the human limbus grew well and reach to confluence in 14 days (Fig. 4B). WST proliferation assay further showed the similar proliferative capacity on days 3, 5 and 7 between the cultures established from central and limbal corneal explants, which was compared with the culture from human explants, among which only limbal explants were growing well (Fig. 4C). Furthermore, clonogenic assay vividly showed that the single cells isolated from murine corneal and limbal epithelia grew well, generated many holoclones, and reached confluence in 10 days on 3T3 feeder layer. In contrast, only single cells isolated from human limbal epithelium, but not from central cornea, were able to generate holoclones and grew to confluence in 14 days (Fig. 4D). These findings provided functional evidence that the murine CESCs with proliferation capacity in regenerating corneal epithelium may be distributed not only in the limbus, but also in the central cornea of murine eye, which is different from human limbal stem cell concept.

## Discussion

Mouse has been widely utilized as experimental models of human diseases in life science and medical research including ocular stem cell and vision research due to their high degree of similarity with human genomes and physiology. For example, CESCs are identified to maintain homeostasis and regeneration of corneal epithelium in both human and mouse corneas. However, their differential localizations of CESCs between human and mouse remain controversial and elusive. Limbal stem cell concept has been recognized and widely accepted in more than two decades^1, 2, 4, 27, 28^. Our previous studies have also provided strong evidence that CESCs reside in limbus of human cornea^14–16, 29^. However, limbal stem cell concept was challenged in differential mammalian species, such as mouse^17^. The present study suggests that the putative corneal stem/progenitor cells may sparsely localized in entire cornea, but not confined to the limbus, in murine cornea.

First, we identified that anatomical murine cornea does not contain a specific structure as a limbal niche for stem cells. The palisades of Vogt are distinctive features of the human corneoscleral limbus, which provide microenvironment niches to harbor and maintain the stemness of human corneal stem cells, and protect the stem cells from physical, chemical or light damage. Numerous evidence from histology, electron microscopy, *in vivo* confocal reflectance microscopy and optical coherence tomography, supports the existence of corneal stem cell niche for human epithelial regeneration from small amount of self-renewing CESC in limbal basal cells. Thus, the palisades of Vogt have been suggested as the reservoir that protects stem cells from traumatic and environmental insults, allows epithelial-mesenchymal interactions, and provides access to chemical signals that diffuse from the rich underlying vascular network^30–32^.

However, controversial findings have been recently realized in other species like mouse. Majo and colleagues demonstrated that murine CESCs were distributed throughout the basal layer of entire corneal epithelium in 2008. Later, Henriksson *et al*. observed that peripheral/limbal corneal epithelium was significantly thinner than central cornea in three strains of mice in 2009^33^. Recently, Zhang *et al*. were able to measure *in vivo* thicknesses of corneal layers in living mice by 3D images using two-photon laser microscopy with fluorescent viability dyes^34^. They observed that the thickness of the entire cornea and corneal epithelium had their maximum at the central cornea, and gradually decreased from peripheral cornea to limbus, which is the thinnest part of corneal epithelium with the thinnest stromal layers in two strains of mice, C57BL/6 and BALB/c. The thickness of limbal epithelial layer is about 20 µm in both strains, which is account for 50 or 37% of thickness of central corneal epithelium in C57BL/6 or BALB/c mice, respectively^34^. The findings were supported with full-field optical coherence microscopy by Grieve *et al*., who detected the palisades of Vogt with limbal crypt features in organ-cultured human corneoscleral rims, but not in fresh mouse cornea, where the limbal region contained only an epithelial trough circumferentially surrounding the cornea^35^.

In the present study, through a series of mouse eye cross sections from different directions, corneal epithelium was further identified to be the thickest in central area while the thinnest at limbal margin, which contains 2–3 layers of epithelial cells without palisades of Vogt-like anatomical structure. These findings suggest that murine limbus does not possess any niche-like features with microenvironment to harbor and protect CESCs.

Second, we identified that the potential corneal epithelial stem/progenitor cells are localized sparsely in the entire mouse cornea including central and peripheral/limbal areas. Although numerous stem cell markers have been proposed, none of these stem/progenitor cells markers have been identified as definitive markers to date^14^. Thus, multiple markers are commonly used to identify the phenotype and potential existence of adult stem/progenitor cells including CESCs as our previous reports^14–16, 29, 36–38^. In this study, five molecular markers, p63, ABCB5, integrin β1, NGF and TrkA, were used to detect the stem/progenitor cells in mouse cornea. The nuclear transcription factor p63, a member of the p53 family, is highly expressed in the basal cells of many human epithelial tissues and the truncated dominant-negative ΔNp63 isoform is the predominant species in stem cells^39^. It was reported that p63 knockout mice lack stratified epithelia, and that p63 expression is associated with proliferative potential in human keratinocytes^12, 40^. ABCB5 is recently proposed stem cell marker, a member of ATP-binding cassette transporters^24^, among which ABCG2 has been identified as a molecular determinant for the side population that enriched in hematopoitic stem cells, and has been proposed as a universal marker for stem cells^41^ including corneal epithelial cells^16^. NGF and its receptor TrkA has been identified to be exclusive localized to a subpopulation of basal limbal epithelial cells where stem cells reside^26^. Integrins are cell surface adhesion molecules. Integrin β1-enriched human epidermal or corneal epithelial cells were demonstrated to possess stem cell property including high colony-forming efficiency and slow-cycling^25, 42^. In the present study, immunofluorescent staining revealed that the cells positively stained with these markers were localized in the basal layer of both limbal and central corneal epithelium, indicating that the corneal stem/progenitor cells may reside in the basal layer throughout entire cornea. Furthermore, the double staining with corneal specific marker K12 and stem cell markers further showed that murine corneal basal epithelial cells that were positive to ΔNp63 and NGF did not express the differentiation marker K12.

Third, we further identified the potential stem cells with slow cycling LRCs in their corneal and limbal location. It has been widely accepted that one of the most reliable ways to identify keratinocyte stem cells takes advantage of the fact that these cells are normally slow-cycling, and hence can be identified experimentally as the so-called LRCs^22, 29, 43–45^. It appears that stem cells have exclusively slow cycling feature to preserve their proliferative capacity and to minimize DNA replication-associated errors while the transient amplifying cells (TACs) could not proliferate for long term. To detect LRCs, one can label all the cells in the epithelium by a repeated or continuous supply of a deoxynucleoside, tritiated thymidine or bromodeoxyuridine (BrdU), followed by a long chase period when the label is lost from all the cycling TACs, so that only stem cells that cycle slowly retain the label, and are designated as LRCs. Using this approach, we detected some BrdU-LRCs localized in the basal epithelial layer of both limbal and central cornea in mice, suggesting that corneal stem cells may be distributed in the entire cornea, not only in limbus.

Finally, we functionally examined the most important stem cells feature, proliferative capacity for tissue regeneration^46–48^. Adult stem cells have been known to have a high proliferative capacity both *in vivo* and *in vitro*; they are essential for the long-term maintenance of normal corneal epithelium, and can be used to reconstitute the entire corneal epithelium in patients with corneal epithelial stem cell deficiencies^16, 48–50^. In the present study, primary corneal epithelial cultures were performed using explants from the central and peripheral/limbal cornea. Results showed that the explants from both limbal and central murine corneas grew well to establish the corneal epithelial cell culture, and could regenerate corneal epithelial sheets when confluent, suggesting that the high colonogenic stem/progenitor cells are possibly located and distributed in entire mouse cornea. This result is different from human cornea, of which only limbal explants grow well to regenerate corneal epithelial sheets, while central corneal explants fail to do so.

In conclusion, this study provides new evidence that the potential corneal epithelial stem cells are reside and sparsely distributed in basal epithelial layer of entire murine cornea, not confined to corneal limbus. Through a series of murine corneal cross-sections from different directions, we identified that anatomically and morphologically the murine limbus is composed of the thinnest epithelium and the thinnest stroma without any palisades of Vogt-like niche structure. Cells expressing five of stem/progenitor cell markers are localized in basal layer of entire murine corneal epithelium. BrdU label-retaining cells, a key characteristic of epithelial stem cells, are detected in both limbal and central cornea of mouse eye. Functionally, corneal epithelium can be regenerated in cultures from central and limbal explants of murine cornea. Such a distribution of mouse CESCs is different from human cornea, where limbal stem cell concept has been well established and accepted to be true. We are aware that new evidence using lineage tracing supports limbal stem cell concept in mouse recently^51^. However, until more definitive data becomes available, the possibility of the existence of stem/progenitor cells outside the limbus should not be excluded. It is important to know that central cornea may provide an alternative source of stem cells when one utilizes mice as animal model for corneal research.

## Materials and Methods

### Animals and Hematoxylin and Eosin (H & E) Staining of Cryosections from Murine Eyes

The animal research protocol for this study was approved by the Institutional Animal Care and Use Committee (IACUC), Center for Comparative Medicine, Baylor College of Medicine. All animals used for experiments were treated in accordance with the Guide for the Care and Use of Laboratory Animals by the National Institute of Health. C57BL/6 mice (6–8 weeks of both sexes) were purchased from The Jackson Laboratory (Bar Harbor, ME).

A series of 8 μm-cryosections of eyeballs were prepared through different directions, radial and tangential, from nasal or temporal to central, as well as from superior or inferior to central cornea using ten eyes of five C57BL/6 mice. The frozen section slides were warmed to room temperature for H & E staining using a routine laboratory protocol.

### Immunofluorescent Staining

Using a previously reported method^14^, 8 µm-cryosections slides of C57BL/6 mice eye tissue were fixed with 4% paraformaldehyde for 10 min and incubated in 10% goat serum/1% BSA/0.3 M glycine in 0.1% PBS-tween for 1 h to block non-specific protein interactions. The sections were then incubated with primary antibodies against mouse ΔNp63, ABCB5, NGF, TrkA, integrin-β1, K14 or K12 overnight at 4 °C. Alexa-Fluor 488 (green) or Alexa-Fluor 594 (red) was used as secondary antibody, and propidium iodide or DAPI was used for nuclear counter staining. The stained slides were photographed with Zeiss laser scanning confocal microscope (LSCM510META, Thornwood, NY).

### ***In vivo*** Detection of BrdU-LRCs in Corneas of C57BL/6 Mice

This animal experiment was performed according to a published method^45^ with modification. In brief, the 4-weeks old C57BL/6 mice were administered by i.p. injections of BrdU (Sigma-Aldrich, 100 mg/kg body weight) daily up to 14 days as the pulse treatment, followed by a chasing period for 4 more weeks. The mouse eyeballs were harvest for the cryosections after chasing. The sections were fixed in cold methanol at 4°C for 10 min, followed by PBS wash for three times. After DNA denatured in 2 N HCL for 30 min, and neutralized in 0.1 M sodium tetraborate buffer for 10 min, the sections were blocked with 1% BSA in PBS for 1 h, and then incubated with anti-BrdU mouse IgG (Abcam, ab6326, 1:100) overnight at 4 °C. Alexa 488 goat anti-rat (1:300) or DAPI were used as secondary antibody or nuclear staining respectively.

### Primary Corneal Epithelial Cells Cultures

Primary corneal epithelial cell cultures were established from explants of corneas. Explants (1 × 1 mm) from murine central cornea or limbus were prepared immediately after euthanasia under a dissecting microscope (Olympus SZ60). Human explants (2 × 2 mm) were prepared from donor corneas within 72 h after death, which obtained from Lions Eye Bank of Texas (Houston, TX). All explants were cultured in 24-well plates with 0.5 ml of supplemented hormonal epidermal medium (SHEM) containing 5% FBS according to previous published methods^15^. Media was changed every two days.

### WST Cell Proliferation Assay

Corneal epithelial cells were established from cultures of central corneal or limbal tissue in 24-well plates. Cells were seeded in a 96-well plate at a density of 10^4^–10^5^ cells/well in 100 µL medium and cultured in a CO_2_ incubator at 37 °C for up to 7 days. Cell proliferation was evaluated by a WST assay kit according to manufacturer’s protocol as previously reported^52^. In brief, each well of cells was added with 10 µl of WST-1 mixture, mixed well and incubated for two hours in an incubator. Before reading the plate, it is important to mix gently on an orbital shaker for one minute to ensure homogeneous distribution of color. Measure the absorbance of each sample using a microplate reader at a wavelength of 450 nm.

### Clonogenic Assays

The clonogenic assay was performed as our previously published method^16, 22^ In brief, The single cells isolated from corneal and limbal epithelia of mouse or human were seeded, in triplicate, at 1×10^3^ cells/cm2 on a feeder layer of 3T3 fibroblast in 6-well culture plates in SHEM medium. The clonal growth capacity was evaluated every other days until confluence in days 10–14 when the cultures were stained with 1% crystal violet.

### Statistical analysis

Student’s t-test was used to compare differences between two groups. One-way ANOVA test was used to make comparisons among three or more groups, followed by Dunnett’s post-hoc test. P < 0.05 was considered statistically significant.
